# Optimizing Nephron Performance: The Old, the New, and the New–Old Diuretic Therapies

**DOI:** 10.3390/biomedicines13061413

**Published:** 2025-06-09

**Authors:** Flavio D. Fuchs, Guilherme S. Procianoy, Leonardo G. Bottino, Sandra C. Fuchs, Paul K. Whelton

**Affiliations:** 1 Division of Cardiology, Hospital de Clínicas de Porto Alegre, Universidade Federal do Rio Grande do Sul, Porto Alegre 90035-903, Brazil; ffuchs@hcpa.edu.br (F.D.F.); gprocianoy@hcpa.edu.br (G.S.P.); lbottino@hcpa.edu.br (L.G.B.); 2 Graduate Program in Cardiology and Cardiovascular Sciences, Universidade Federal do Rio Grande do Sul, Porto Alegre 90035-003, Brazil; 3 Departments of Epidemiology and Medicine, Tulane University, New Orleans, LA 70112, USA; pkwhelton@gmail.com

**Keywords:** nephrons, diuretics, blood pressure, hypertension, heart failure, chronic kidney disease

## Abstract

Pharmacological influence on nephron function has modified the clinical course of hypertension, heart failure, and chronic kidney disease. (CKD). This is a review of the efficacy of old diuretics and the incremental efficacy of new diuretics in managing hypertension, heart failure, and CKD, concluding with new evidence on the effectiveness of old agents. The efficacy of “older” diuretic agents, such as thiazide and loop diuretics, on heart failure and CKD has been primarily explored in nonrandomized studies. However, the efficacy of these agents and indapamide, a slightly newer but still “old” diuretic in preventing blood pressure-related cardiovascular disease, has been demonstrated in randomized controlled trials. Potassium-sparing agents counteract some of the adverse effects of thiazides and have been shown to prevent cardiovascular events in patients with heart failure. Newer drugs with a diuretic effect, such as gliflozins, act through a new mechanism of action in the kidney and have shown efficacy in controlling symptoms and preventing cardiovascular events in patients with heart failure, regardless of diabetes. Furthermore, gliflozins have prevented the progression of chronic kidney disease in patients with and without diabetes mellitus. New evidence detailing the efficacy of old agents has emerged. Chlorthalidone had a large blood-pressure-lowering effect in patients with stage IV CKD. Acetazolamide was effective in accelerating the clinical control of patients with acute heart failure, including patients with some reduction in kidney function. We anticipate investigating the comparative impact of combining different agents to optimize nephron function in the future.

## 1. Introduction

**Figure 1:**
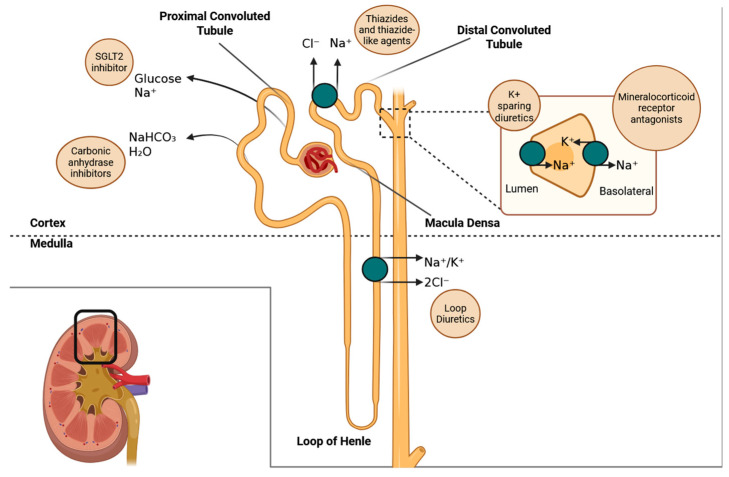
Sites in the nephron at which different agents have a diuretic effect.

Diuretics have been in use for more than seven decades and include thiazide and thiazide-like diuretics, loop diuretics, potassium-sparing diuretics, and acetazolamide. Gliflozins (sodium-glucose co-transporter 2–[SGLT2] inhibitors) and newer mineralocorticoid receptor antagonists, such as finerenone and eplerenone, are more recent additions to this group. Each class of diuretic targets specific sites within the nephron, as illustrated in Figure 1. Table 1 describes how each class of diuretics exerts its effects, their specific molecular mechanisms, along with their site of action, primarily targeting transporters or receptors located along different segments of the nephron, and pharmacological effects:

- Thiazide diuretics, such as hydrochlorothiazide and indapamide, function primarily in the distal convoluted tubule of the kidneys. They inhibit the sodium/chloride co-transporter (NCC), which leads to a reduction in the reabsorption of sodium and chloride. As a result, this causes a decrease in extracellular volume and lowers arterial pressure. Over time, their antihypertensive effect is further enhanced by a reduction in vascular resistance.
- Mineralocorticoid receptor antagonists, such as spironolactone, eplerenone, and finerenone, work by blocking aldosterone from binding to its receptor in the collecting duct of the kidneys. This action prevents the transcription of sodium channels and sodium/potassium-ATPase pumps, which leads to reduced sodium retention and decreased potassium excretion. Additionally, finerenone has effects in non-renal tissues, particularly in the heart, where it influences gene expression related to fibrosis and inflammation.
- Gliflozins, SGLT2 inhibitors such as dapagliflozin and empagliflozin, work by blocking the SGLT2 transporter located in the proximal tubule of the kidneys, which prevents the reabsorption of glucose and sodium. As a result, this action causes osmotic diuresis (increased urine output due to the presence of certain substances in the urine) and natriuresis (excretion of sodium in the urine). This process restores tubuloglomerular feedback, reduces glomerular hyperfiltration, and ultimately lowers glomerular pressure and wall stress. Additionally, these medications have metabolic and anti-inflammatory effects that contribute to cardiovascular and renal protection.
- Acetazolamide works primarily in the proximal tubule of the kidney, where it inhibits the enzyme carbonic anhydrase. This enzyme is responsible for converting carbonic acid into water and carbon dioxide (CO_2_). By inhibiting this process, acetazolamide decreases the availability of protons (H^+^), which impairs the Na^+^/H^+^ exchanger. As a result, bicarbonate reabsorption is reduced, leading to urine alkalinization and a mild diuretic effect. This medication is particularly beneficial for enhancing diuresis in patients experiencing fluid overload, especially when used in combination with loop diuretics.

The kidneys perform several functions beyond their primary role in filtering waste metabolites, including regulating body fluids, maintaining electrolyte balance, and controlling blood pressure (BP). These nephron-mediated functions can be enhanced pharmacologically using diuretics to treat hypertension and heart failure. Furthermore, the use of diuretics to augment tubular function within the extant nephrons of patients with glomerulosclerosis constitutes a key strategy for preserving kidney function. Strictly, diuretics act primarily by blocking the normal nephron mechanisms of sodium reabsorption, but the net effect is beneficial for the management of hypertension, heart failure, and chronic kidney disease (CKD).

**Table 1:** Site of action and pharmacological effects of key diuretic classes.

Drug Class	Primary Site of Action	Mechanism of Action	Pharmacological Effects
Thiazides	Distal convoluted tubule	Inhibit Na^+^/Cl^−^ co-transporter (NCC)	↓ Sodium reabsorption ↓ Intravascular volume ↓ Cardiac preload
Mineralocorticoid receptor antagonists (spironolactone, finerenone)	Collecting duct and cardiomyocytes	Block aldosterone binding at mineralocorticoid receptors	↓ Sodium reabsorption ↓ Inflammation ↓ Fibrosis
Gliflozins (SGLT2 inhibitors)	Proximal convoluted tubule	Inhibit sodium-glucose co-transporter 2 (SGLT2)	↓ Glomerular pressure ↓ Wall stress Improved metabolic and hemodynamic profile
Acetazolamide	Proximal convoluted tubule	Inhibit carbonic anhydrase → ↓ H^+^ availability for Na^+^/H^+^ exchanger	↓ Bicarbonate reabsorption ↑ Urine alkalinization Mild diuresis

In addition to discovering the diuretic effects of gliflozins, new insights regarding established diuretics have emerged. Randomized controlled trials (RCTs) have demonstrated the efficacy of chlorthalidone [1,2] and acetazolamide [3] for the treatment of hypertension and inducing diuresis among patients with advanced chronic kidney disease (CKD) and heart failure, respectively. This broadening array of diuretic options, acting at the various sites in the nephron depicted in the figure, holds promise for optimizing nephron function and introducing innovative pathways for managing heart failure, CKD, and hypertension. In this review, we present evidence detailing the efficacy of established agents (the old), newly discovered agents (the new), and established agents with newfound applications (the new–old). The review focuses on the period of discovery of the agents, detailing their efficacy in hypertension, heart failure, and chronic kidney disease.

## 2. The Old: Established Diuretics

The discovery of thiazide diuretics and chlorthalidone, a thiazide diuretic, during the 1950s was a milestone in pharmaceutical development. Previously available pharmaceuticals, such as mercurial diuretics and carbonic anhydrase inhibitors, chiefly acetazolamide, exhibited only modest diuretic efficacy and were poorly tolerated [4]. Loop diuretics were introduced during the 1960s [5]. Subsequently, indapamide, another thiazide-like diuretic, was approved for clinical use. In this review, we utilize the term “thiazides” to encompass both thiazide and thiazide-like agents, given their common mechanism of action in the distal convoluted nephron. The introduction of potassium-sparing agents, capable of pharmacologically antagonizing aldosterone’s actions [6,7,8], further enhanced the range of diuretic options.

The primary initial application of diuretics was in patients with heart failure and CKD, although their comparative effects against placebo were not explored in RCTs under these conditions. A pivotal turning point emerged in 1958 when a seminal case series revealed the efficacy of chlorothiazide in managing hypertension [9]. Subsequently, various thiazide and thiazide-like diuretics were assessed for their effect on BP [10]. RCTs also shed light on the impact of potassium-sparing agents. The concurrent administration of thiazide diuretics with potassium-sparing agents promotes a more pronounced reduction in BP, concomitant with the attenuation of hypokalemia and hyperglycemia [11].

Old agents were tested in many landmark RCTs that proved their efficacy for lowering BP and preventing cardiovascular disease (CVD) in patients with hypertension. Hydrochlorothiazide was part of the triple therapy tested in two seminal RCT reports from an RCT conducted by the Veterans Administration Cooperative Study Group on Antihypertensive Agents. The first report, published in 1967, demonstrated the dramatic benefit of triple-therapy drug treatment (fixed-dose hydrochlorothiazide and reserpine combination pill plus hydralazine given as a separate pill) compared with placebo in a subgroup of adults who had an average baseline diastolic BP between 115 and 129 mmHg [12]. The second report, published in 1970, demonstrated the cardiovascular benefits of the same treatment comparison in the remaining group of adults with an average baseline diastolic BP between 90 and 114 mmHg [13]. The Medical Research Council (MRC) Working Party trial in older adults, published in 1992, showed that treatment using a combination of hydrochlorothiazide with amiloride reduced the incidence of stroke and coronary heart disease events in older adults [14]. The 1991 Systolic Hypertension in the Elderly Program [15] study demonstrated that the treatment of isolated systolic hypertension in older adults with a chlorthalidone-based regimen markedly reduced the incidence of stroke (primary outcome), heart failure, and CVD compared with placebo [15].

The HYVET trial [16], which was conducted in patients who were 80 years or older, and the Progress trial, which was conducted in patients who had a history of stroke [17], employed an indapamide-based intervention. The HYVET was the first trial to demonstrate a reduction in all-cause deaths. The Progress trial demonstrated a substantial reduction in stroke recurrence.

In a double-blind comparison between two antihypertensive treatments, the INSIGHT trial demonstrated that the participants randomized to a calcium channel blocker (long-acting nifedipine) or a combination of hydrochlorothiazide and amiloride had a similar incidence of CVD events but the diuretic group had a lower incidence of fatal myocardial infarction compared with their counterparts who were randomized to nifedipine [18]. The Antihypertensive and Lipid-Lowering to Treat Heart Attack Trial (ALLHAT) was the largest RCT to compare first-step therapy of hypertension with a long-acting diuretic (chlorthalidone), a calcium channel blocker (amlodipine), or an angiotensin-converting enzyme inhibitor (lisinopril) for the prevention of coronary heart disease (primary outcome) and other CVD events [19]. The incidence of coronary heart disease (primary outcome) and all-cause mortality was similar to the treatment with the three agents. Still, chlorthalidone was more effective for preventing heart failure, especially compared with amlodipine, and was slightly more effective than lisinopril for preventing stroke in secondary analyses.

The Systolic Blood Pressure Intervention Trial (SPRINT) was designed to compare adults with hypertension at increased risk for CVD who were randomized to a systolic BP target of less than 120 mm Hg (intensive treatment) or 140 mm Hg (standard treatment). Compared to standard treatment, the intensively treated group had an average reduction of 27% in the incidence of the primary outcome (composite CVD event), and 25% for all-cause mortality [20]. The individual components of the composite of CVD events, such as myocardial infarction, heart failure, and CVD mortality, were all significantly reduced in the intensively treated group. The use of diuretics was more frequent in the intensive (55%) compared to the standard treatment arm (33%).

Except for spironolactone, which demonstrated its effectiveness in preventing CVD events in patients with a reduced ejection fraction but not a preserved ejection fraction, older diuretic agents have not undergone RCTs to assess their impact on preventing major CVD events in patients with heart failure. However, the substantial reduction in incident heart failure in many trials of antihypertensive therapy in older adults, including a reduction of 54% in the SHEP, 64% in the HYVET, and 63% in the SPRINT, provides strong indirect evidence that diuretics are beneficial for the prevention of heart failure in patients with hypertension. Spironolactone is also used to manage hypertension, particularly in patients with primary aldosteronism, and as a fourth-line treatment in those with resistant hypertension. The efficacy of traditional diuretic agents in preventing the progression of CKD and the occurrence of CVD in patients with CKD has not been investigated in RCTs. In the SPRINT patients with and without CKD, there was a reduction in the incidence of the primary outcome composite CVD, similarly to the entire trial, and a reduction in proteinuria in the intensively treated group compared with the standard treatment group [20,21]. However, the trial was not designed to directly compare the efficacy of different BP agents. Table 2 summarizes the key randomized controlled trials discussed in this paper.

ACE inhibitors and angiotensin receptor blockers (ARBs) are recognized as BP-lowering medications that are particularly effective in supporting the preservation of kidney function in those with heavy proteinuria or kidney disease. Despite blocking angiotensin receptors along the nephron, the tendency to promote natriuresis is typically masked by their blood-pressure-lowering effects.

## 3. The New: Recently Introduced Agents

Sodium-glucose cotransporter 2 inhibitors (SGLT2i), also known as gliflozins (hereafter referred to as gliflozins), provide a novel approach to managing cardiovascular and renal disease [22]. Originally developed to treat diabetes, these agents exhibited a dose-dependent glycosuric effect [23] and demonstrated a capacity to lower blood glucose levels [24]. When used in conjunction with metformin, as a second-line treatment, they resulted in more effective glycemic control [25,26].

In line with FDA requirements to investigate the effectiveness of hypoglycemic treatments in reducing CVD events, gliflozins were initially assessed in patients with diabetes [25,27], and subsequently in patients without diabetes [28,29], with heart failure [30,31,32,33], and with CKD [29]. The treatment outcomes in these RCTs were remarkable. For instance, Empagliflozin reduced the incidence of death from any cause by 32% in patients with diabetes [31]. In comparison, Dapagliflozin treatment resulted in a 48% reduction in all-cause mortality in patients with mild-to-moderate CKD and no diabetes [29]. In patients with heart failure and reduced ejection fraction, Dapagliflozin led to a 17% decrease in mortality [30].

In an RCT involving participants with diabetes and albuminuric CKD, canagliflozin reduced the risk of progression to end-stage kidney disease by 32% [34]. Similarly, dapagliflozin reduced the risk of progression to end-stage kidney disease by 36% in patients with and without diabetes [28]. Notably, gliflozins were the first diuretic medications to demonstrate a beneficial impact on CKD progression in RCTs.

The finding that gliflozins produce similar CVD prevention benefits in patients with and without diabetes strongly suggests that their protective mechanisms cannot be attributed solely to the glucose-lowering effects of these agents. Several hypotheses have been proposed to explain the protective effects, such as increased ATP production [35], the inhibition of the sodium-hydrogen exchange, which could protect both cardiomyocytes and kidney cells [36,37], and reduced inflammation in the heart and endothelium [38,39,40]. Diverse mechanisms have been proposed to explain how gliflozins exert protective CVD and kidney effects.

The most consistent mechanisms behind the CVD protective effect of gliflozins seem to be natriuresis and osmotic diuresis, activated by increased salt delivery to the macula densa. Initially, their BP-lowering effect was observed alongside glucose reduction in RCTs. A meta-analysis of 27 studies that pooled experience in 12,960 study participants reported a systolic BP reduction of 4.0 mmHg (95% CI, 3.5 to 4.4) with gliflozins [41]. Some larger trials demonstrating CVD event prevention with gliflozins did not report BP effects [32,42]. Others reported variable BP effects ranging from 1.2 mmHg [33] to 7.5 mmHg [43].

The BP-lowering effect described in RCT that compared a gliflozin to a placebo cannot serve as conclusive evidence for the magnitude of BP reduction that can be achieved with gliflozin treatment because trial participants randomized to the placebo group may have increased their use of other antihypertensive medications. Furthermore, most of these trials lacked a comprehensive protocol for standardized BP measurement and ambulatory BP monitoring (ABPM). Likewise, many of the RCTs included many non-hypertensive trial participants.

The RCTs that have directly compared gliflozins with a placebo or with other BP medications provide the most meaningful estimates of the extent to which gliflozins can lower BP, especially the trials that have employed ABPM. The six RCTs that met these criteria were included in a meta-analysis conducted by Baker et al. [44]. The trials included had a treatment duration of 4 to 12 weeks and enrolled 1259 participants. The net reduction in 24 h systolic BP compared to placebo was 3.8 mmHg (95% CI 4.2 to 2.3) [44]. This BP-lowering effect is not so intense and probably would, at best, reproduce the effects of low doses of diuretics in patients with hypertension. The beneficial effect in patients with heart failure and CKD may be derived from other actions in these patients.

In an RCT focused on black participants with hypertension and diabetes, empagliflozin (25 mg) exhibited an unusual BP-lowering effect. Systolic and diastolic BP decreased progressively during 24 weeks of treatment. At week 12, the net difference in systolic BP compared to placebo was 5.2 mm Hg [95% CI, 1.2–9.2]. By week 24, the systolic BP reduction had increased to 8.4 mmHg [95% CI, 3.0–13.7] [45]. If this BP-lowering effect were to be sustained over the long term, it could explain much of the CVD prevention described in the gliflozin trials [46].

These findings underscore that the cardiovascular and renal benefits of SGLT2 inhibitors likely extend beyond modest BP-lowering effects. Indeed, the modest systolic BP reductions observed in ABPM-based meta-analyses (approximately 3.8 mmHg) suggest that other mechanisms, such as improved tubuloglomerular feedback, reduction in intraglomerular pressure, and anti-inflammatory effects, play a pivotal role. Finerenone, a selective nonsteroidal mineralocorticoid receptor antagonist (MRA), is a relatively new diuretic. As the drug class name implies, finerenone and other MRAs work by blocking the receptor for the hormone aldosterone, which results in diuresis, the retention of potassium, and lowering BP. The possibility that finerenone, like other MRA antagonists, exerts its effects through the action in non-renal receptors, such as cardiomyocytes, may also be considered. Two large RCTs have demonstrated that finerenone improves nephron function and prevents CKD progression and major CVD events in selected patients with CKD, diabetes mellitus, and albuminuria [47,48]. Unlike gliflozins, which have a new mechanism of action, finerenone reproduces the effects of other mineralocorticoid receptor antagonists, such as spironolactone and eplerenone. These older agents have not been tested in RCT with sufficient power to assess their nephron-protective effect. In patients with diabetes mellitus and CKD with proteinuria, the incidence of a primary outcome event occurred (12.4%) in the finerenone group and 4.2% in the placebo group (HR 0.87, 95% CI 0.76 to 0.98), with the benefit-driven primarily by a lower incidence of hospitalization for heart failure [47]. A sustained decrease of at least 40% in the eGFR from baseline, a secondary outcome, was also reduced by treatment with finerenone (HR 0.81, 95 CI 0.72–0.92) [48]. A recent randomized controlled trial showed that finerenone reduced the rate of worsening heart failure events in patients with a mildly reduced or preserved ejection fraction (HR 0.82 95% CI 0.71–0.94) [49].

## 4. The New–Old: Rediscovered Utility of Traditional Agents

Early studies did not identify a diuretic effect of thiazides in patients with a reduced GFR [50,51]. In subsequent years, relatively small, non-randomized studies suggested that thiazides had a modest diuretic effect in patients with a reduced GFR [52,53,54,55,56,57]. Even the first RCTs that explored the effect of thiazides or thiazides in combination with loop diuretics in patients with CKD had design limitations and small sample sizes that precluded definitive conclusions about the effect of thiazide therapy in patients with CKD [58,59,60,61,62]. The absence of compelling evidence for a clinically useful diuretic effect of thiazide in patients with advanced CKD and the concurrent knowledge that loop diuretics had a strong diuretic effect in such patients resulted in the almost exclusive use of loop diuretics for diuresis in patients with advanced CKD.

Two uncontrolled interventional studies suggested that thiazide diuretics could be effective in patients with low GFR [1,63]. These studies led to a recent high-quality RCT investigation of the efficacy of thiazide-like diuretic chlorthalidone in patients with advanced CKD [2]. The unexpected results of this RCT may be characterized as a Black Swan phenomenon. The trial participants (81 randomized to chlorthalidone and 79 to placebo) had uncontrolled hypertension based on ABPM and stage 4 CKD. During the trial, the dose of chlorthalidone could be titrated from 12.5 mg initially to a maximum of 50 mg, with an average chlorthalidone dose of 23.1 mg at the end of the trial. The net ambulatory 24 h systolic BP treatment effect (chlorthalidone minus placebo group difference from baseline to 12 weeks) was 10.5 (95% CI 6.4 to 14.6) mm Hg and the corresponding net difference for nighttime systolic BP was 11.1 (5.6 to 16.6) mm Hg. The effect was similar in furosemide users and nonusers. Urinary albumin-to-creatinine ratio was significantly decreased in the participants treated with chlorthalidone compared with placebo. In a subsample of participants with resistant hypertension, there was a 24 h ABPM reduction of 13.9 (8.4 to 19.4) mm Hg in the chlorthalidone group compared with placebo [64].

The combined use of thiazide and loop diuretics was more common in patients with heart failure, despite the absence of a demonstration of synergism in RCT. In a small short-term RCT, the addition of hydrochlorothiazide (50 mg) increased weight loss compared to placebo by 0.73 kg/day in patients with acute heart failure [65]. In an RCT with a similar design but a larger sample size and dosing of hydrochlorothiazide based on the patient level of GFR (25 to 100 mg), the adjusted placebo-controlled reduction in weight on the third day was 1.14 kg (95% CI 0.42 to 1.84 kg). There was no effect on dyspnea [66].

Acetazolamide, a carbonic anhydrase inhibitor that reduces proximal tubular sodium reabsorption, is another old diuretic that may have a greater diuretic effect than previously recognized. In patients with acute heart failure (most with a GFR below 60 mL/min/1.73 m^2^), the random assignment to intravenous acetazolamide, 500 mg once daily, (n = 259) was compared with placebo (n = 260) [3]. All of the participants were being treated concurrently with furosemide. The successful diminution of edema within 3 days (primary endpoint) occurred in 42.2% of the patients who had been randomly assigned to treatment with acetazolamide and 30.5% of those randomized to placebo (*p* < 0.001). At discharge, 78.8% of the participants were randomized to acetazolamide, compared to 62.5% of participants in the placebo arm who experienced decongestion.

In light of the importance of safety in clinical decision making, Table 3 presents a comparative summary of the most common adverse effects and safety considerations for each class of diuretic agents. Although the efficacy of these medications has been extensively studied, their use must be carefully weighed against potential risks, especially in patients with CKD. Thiazides and loop diuretics may lead to significant electrolyte disturbances, including hypokalemia and hyponatremia. Mineralocorticoid receptor antagonists, such as finerenone, are associated with hyperkalemia, especially in patients with impaired renal function. SGLT2 inhibitors are generally well tolerated but carry specific risks, including genital mycotic infections, volume depletion, and, in rare cases, euglycemic ketoacidosis. Acetazolamide may cause metabolic acidosis and paresthesia. Awareness of these safety profiles is essential for optimizing nephron function through individualized and risk-adjusted pharmacologic strategies.

## 5. Clinical Integration and Future Directions

Developing drugs that enhance nephron function represents a significant advancement in the management of non-communicable diseases. The 1950s and 1960s witnessed the first wave of discoveries, during which numerous drugs targeting the function of the nephron were introduced. The second wave has been marked by the discovery and introduction of gliflozins and the recognition of unexpected beneficial effects of the “old” diuretics.

We anticipate an investigation into the comparative impact of combining different agents to optimize nephron function in the future. Without such studies, the strategic combination of agents targeting distinct nephron segments, guided by clinical responses, will likely emerge as a valid approach for optimizing nephron performance.

## Figures and Tables

**Table 2:** A summary of the principal randomized controlled trials discussed.

Trial Name	Population	Intervention	Comparator	Primary Outcome(s)	Main Result
SHEP (Systolic Hypertension in the Elderly Program, 1991) [15]	Older adults with isolated systolic hypertension	Chlorthalidone-based therapy	Placebo	Stroke	↓ Stroke by 36%, ↓ heart failure by 54%
MRC Trial (Medical Research Council, 1992) [14]	Older hypertensives	HCTZ + amiloride	Placebo	Stroke, CHD	↓ Stroke and CHD with diuretic combination
INSIGHT (International Nifedipine GITS Study: Intervention as a Goal in Hypertension Treatment, 2000) [18]	Hypertensives	Nifedipine GITS vs. hydrochlorothiazide + amiloride	Head to head	CVD outcomes	Similar total CVD; ↓ fatal MI with diuretic
PROGRESS (Perindopril Protection Against Recurrent Stroke Study, 2001) [17]	Stroke survivors	Perindopril ± indapamide	Placebo	Recurrent stroke	↓ Stroke recurrence by 28% (43% with combo)
ALLHAT (Antihypertensive and Lipid-Lowering Treatment to Prevent Heart Attack Trial, 2002) [19]	High-risk hypertensives (n ≈ 33,000)	Chlorthalidone vs. amlodipine or lisinopril	Head to head	CHD, stroke, heart failure	Chlorthalidone superior for heart failure; similar CHD/mortality
Treat Heart Attack Trial (ALLHAT) [19]	Same as above	See ALLHAT	See ALLHAT	Coronary heart disease	↓ HF with diuretic, no superiority for CHD
HYVET (Hypertension in the Very Elderly Trial, 2008) [16]	Adults ≥80 years with hypertension	Indapamide ± perindopril	Placebo	Stroke, all-cause mortality	↓ Stroke risk by 30%, ↓ all-cause mortality by 21%
SPRINT (Systolic BP Intervention Trial, 2015) [20]	High-risk hypertensives	SBP target <120 mmHg	SBP target <140 mmHg	CVD events, mortality	↓ CVD events by 25%, ↓ mortality by 27%

**Table 3:** Comparative safety profile of diuretic drug classes.

Drug Class	Common Adverse Effects	Considerations in CKD
Thiazide diuretics	Hypokalemia, hyperuricemia, mild hyperglycemia, hyponatremia	Reduced efficacy at GFR <30 mL/min, risk of hyponatremia
Loop diuretics	Hypokalemia, hypomagnesemia, dehydration, ototoxicity (high doses)	Still effective in advanced CKD, monitor electrolytes closely
Mineralocorticoid receptor antagonists (e.g., spironolactone, finerenone)	Hyperkalemia, gynecomastia (spironolactone), risk of worsening kidney function in advanced CKD	Monitor potassium and eGFR; finerenone safer than spironolactone in some CKD contexts
SGLT2 inhibitors (gliflozins)	Genital mycotic infections, volume depletion, rare ketoacidosis, risk of urinary tract infections	Effective down to eGFR ~20–25 mL/min; monitor volume status and euglycemic DKA risk
Acetazolamide	Metabolic acidosis, hypokalemia, fatigue, paresthesia, renal stone formation (rare)	Use with caution; can cause acidosis in advanced CKD
